# Characterization of diffuse lung function in children with *Mycoplasma pneumoniae* pneumonia

**DOI:** 10.3389/fped.2024.1443877

**Published:** 2025-01-06

**Authors:** Li Wang, Qianqian Li, Jie Hu, Ronghua Luo, Yaping Duan, Tao Ai

**Affiliations:** Pediatric Respiratory Medicine Department, Chengdu Women’s and Children’s Central Hospital, School of Medicine, University of Electronic Science and Technology of China, Chengdu, China

**Keywords:** pneumonia, *Mycoplasma pneumoniae*, lung diffusion function, children, DLCO

## Abstract

**Background:**

*Mycoplasma pneumoniae* infection accounts for a high proportion of community-acquired pneumonia and the incidence rate of severe *M. pneumoniae* pneumonia (MPP) has increased year by year. This study investigated the changes in lung diffusion function after *M. pneumoniae* infection, compared the lung diffusion and ventilation function of children with mild (MMPP) or severe *M. pneumoniae* pneumonia (SMPP) infections, and explored their clinical significance.

**Objective:**

To study the changes in pulmonary ventilation and pulmonary diffusion function in children with MPP, and explore their clinical significance.

**Methods:**

Data from 97 children with *M. pneumoniae* pneumonia hospitalized in Chengdu Women and Children's Central Hospital from June 2023 to December 2023 were collected and the participants were divided into an MMPP group (*n* = 44) and an SMPP group (*n* = 53). The changes in pulmonary ventilation function and diffusion function were compared between the two groups.

**Results:**

The *Z*-scores of forced vital capacity and forced expiratory volume in the first second in the SMPP and MMPP groups were −1.684 ± 0.902 and −1.986 ± 0.818, and 0.164 ± 1.795 and −0.6104 ± 1.276, respectively. In the SMPP group, the two aforementioned indicators were lower than the normal value and significantly lower than those in the MMPP group (*P* < 0.001). The carbon monoxide diffusion capacity in the SMPP group (−5.931 ± 0.827) was significantly lower than that in the MMPP group (−5.0775 ± 1.1134) (*P* < 0.001). The forced expiratory flow at 75% vital capacity and the maximum mid expiratory flow in the SMPP group were −2.006 ± 1.2582 and −1.878 ± 1.008, respectively, which were lower than the normal value.

**Conclusion:**

SMPP results in more severe ventilation dysfunction and diffuse dysfunction than MMPP.

## 1 Introduction

*Mycoplasma pneumoniae* pneumonia (MPP) refers to pulmonary inflammation caused by an *M. pneumoniae* (MP) infection, which can affect bronchi, bronchioles, alveoli, and the pulmonary interstitium. According to MPP’s clinical classification, it can be divided into mild (MMPP), severe (SMPP), and fulminant *M. pneumoniae* pneumonia (FMPP) (1–3). In mild cases, the alveolar spaces are mostly infiltrated by neutrophils, while in severe cases, there is infiltration by lymphocytes, plasma cells, and macrophages; thickening and edema of the alveolar walls; exudation of fibrin and polypoid tissue in the alveolar cavity; necrosis and shedding of epithelial cells in the bronchus and bronchioles; destruction of cilia; and proliferation of fibroblasts, resulting in fibrotic changes and thus affecting gas exchange (2). Approximately 10% of children with COVID-19 have diffusion dysfunction after 3 months of follow-up, and the decline is more obvious in children with severe symptoms (4). However, one study has shown that carbon monoxide diffusion volume (DLCO) can return to normal 6 months after pneumococcal pneumonia in children (5). The improvement in pulmonary function examinations in children with MPP is of great significance for monitoring disease outcomes and improving the prognosis (6, 7). Previous studies have shown that pulmonary ventilation dysfunction in children with MPP can manifest as obstructive, restrictive, and mixed types (6, 8). However, there is no relevant study on whether pulmonary ventilation dysfunction in children with MMPP is different from that in children with SMPP, and there are relatively few studies on the changes in diffusion pulmonary function of children with MPP of different severities. Understanding the differences in lung function and diffuse lung function indicators between mild and severe *M. pneumoniae* pneumonia is beneficial for monitoring the severity of the disease and disease prognosis in clinical practice and providing guidance for disease diagnosis and treatment. This study retrospectively analyzed data from 97 children diagnosed with *M. pneumoniae* pneumonia, compared the ventilation lung function and diffusion lung function-related indicators in children with MMPP and SMPP, and further explored the changes in lung function in the children infected with *M. pneumoniae*.

## 2 Methods

### 2.1 Study population

The data from 97 children (aged 6–13 years) with MPP who were hospitalized in the Children's Respiratory Department of Chengdu Women's and Children's Central Hospital from June 2023 to December 2023 were collected from electronic records by a researcher according to the Chinese “Guidelines for the Diagnosis and Treatment of *Mycoplasma Pneumoniae* in Children (2023 Edition)” (2). They were divided into an MMPP group and an SMPP group. The studies involving human participants were reviewed and approved by the ethics board of Chengdu Women and Children Center Hospital (Ethical number: [2021]203). Written informed consent to participate in this study was provided by the participants’ legal guardians.

The inclusion criteria for MMPP were according to clinical manifestations, chest imaging changes, and pathogenic examination as follows: (1) MP antibody titer of single serum ≥1:160 [particle agglutination (PA) method] or, during the course of the disease, the MP antibody titer in double serum increased four-fold or more; (2) positive for MP DNA or RNA (through a throat swab examination); (3) failed to reach SMPP diagnostic thresholds.

The inclusion criteria for SMPP were those who met one of the following criteria: (1) persistent high fever (above 39°C) ≥5 days or fever ≥7 days, with no downward trend of heat peak; (2) suffering from wheezing, shortness of breath, dyspnea, chest pain, hemoptysis, etc.; (3) experiencing extrapulmonary complications but the criteria for critical illness are not met; (4) in a resting state, inhaled air results in a pulse oxygen level ≤0.93; (5) presenting with one of the following imaging manifestations: (i) a single lung lobe ≥2/3 is involved with uniform high-density consolidation or two or more lung lobes have high-density consolidation (regardless of the size of the involved area), which can be accompanied by moderate to large pleural effusion and can also be accompanied by localized bronchiolitis; (ii) single lung diffuse or bilateral lobar bronchiolitis ≥4/5, which can be combined with bronchitis, and there is the formation of mucus plug leading to atelectasis; (6) the clinical symptoms worsen progressively, and the imaging shows that the range of lesions progressed more than 50% within 24–48 h; (7) C-reactive protein (CRP), lactate dehydrogenase (LDH), and/or D-dimer levels increase significantly.

The exclusion criteria were as follows: (1) Children with chronic inflammation of the respiratory system, bronchial asthma, immune system defects or suppression, chronic diseases of other organs, etc.; (2) children who had recently been treated with immunomodulators or inhibitors; (3) children with underlying diseases or major diseases; (4) children who met the diagnostic criteria of critical *M. pneumoniae* infection according to the “Guidelines for the Diagnosis and Treatment of *Mycoplasma Pneumoniae* in Children (2023 Edition)” as critical children may have unstable vital signs that make it difficult to cooperate with lung function tests and may also have other comorbidities that could affect the results of this study; (5) children with incomplete medical records.

### 2.2 Methods

The clinical manifestations, laboratory examinations, imaging characteristics, and pulmonary function tests, including forced spirometry, lung volume measurement, and lung carbon monoxide diffusion capacity tests, of children were retrospectively analyzed. The laboratory examinations included CRP, LDH, D-dimer, and other indicators.

### 2.3 Pulmonary function measurement

The pulmonary ventilation function of the children was measured using a MasterScreen Pulmonary Function Testing System produced by Jaeger in Germany. The measured parameters were forced vital capacity (FVC), forced expiratory volume in the first second (FEV1), forced expiratory flow at 25%, 50%, or 75% vital capacity (FEF25, FEF50, or FEF75), maximum mid expiratory flow (MMEF), functional residual capacity (FRC), total lung volume (TLC), residual volume (RV), residual to total lung volume ratio (RV/TLC), and DLCO [hemoglobin (Hb) correction was performed for anemic patients]. The above data were converted to *Z*-scores using the Global Lung Initiative reference equation.

### 2.4 Statistical analysis

Data analysis was performed using SPSS 26.0 statistical software. If the quantitative data followed a normal distribution (*x* ± *s*), two independent sample *t*-tests were used for inter-group comparisons. Quantitative data that were not normally distributed were described by the median and upper and lower quartiles (M, P25–P75), and compared between groups using the rank sum test of two independent samples (Mann–Whitney *U* test). Categorical data were described by percentages and compared between groups by the Chi-square test. *P* < 0.05 was considered statistically significant.

## 3 Results

### 3.1 Comparative demographic information

There were 44 children in the MMPP group, with 24 boys and 20 girls aged 6–13 years. There were 53 cases in the SMPP group, with 27 boys and 26 girls aged 6–13 years. There was no statistical difference in sex and age composition ratio (*P* > 0.05), as shown in Table 1.

**Table 1:** Comparison of the *Z*-scores transformed from pulmonary ventilation function and lung diffusion function indexes in the SMPP and MMPP groups.

Indicator	SMPP group (*n* = 53)	MMPP group (*n* = 44)	*t*	*P*
Age	8.88 ± 1.96	8.22 ± 1.75	−1.524	0.084
Male %	54.55	50.94	0.512	0.493
FEV1	−1.986 ± 0.818	−0.6104 ± 1.276	−5.748	0.000
FVC	−1.684 ± 0.902	0.1642 ± 1.795	−5.798	0.000
FEV1/FVC	−0.998 ± 0.925	−1.3783 ± 1.083	1.700	0.093
MMEF	−1.878 ± 1.008	−1.4915 ± 1.157	−1.604	0.113
FEF75	−2.006 ± 1.258	−1.5294 ± 1.019	−1.890	0.062
DLCO	−5.931 ± 0.827	−5.0775 ± 1.113	3.906	0.000
FRC	−0.773 ± 0.841	−0.1144 ± 0.795	−3.644	0.000
TLC	−1.223 ± 0.935	−0.1151 ± 1.014	−5.128	0.000
RV	0.115 ± 0.523	0.3534 ± 0.525	−2.056	0.043
RV/TLC %	1.162 ± 0.664	1.0109 ± 0.595	1.083	0.282

MMPP group, mild *Mycoplasma pneumoniae* pneumonia group; SMPP group, *severe M. pneumoniae* pneumonia group; FEV1, forced expiratory volume in the first second; FVC, forced vital capacity; MMEF, maximum mid expiratory flow; FEF75, forced expiratory flow at 75% vital capacity; DLCO, carbon monoxide diffusion volume; FRC, functional residual capacity; TLC, total lung volume; RV, residual volume; RV/TLC, residual to total lung volume ratio.

### 3.2 Comparison of pulmonary ventilation function

**Figure 1:**
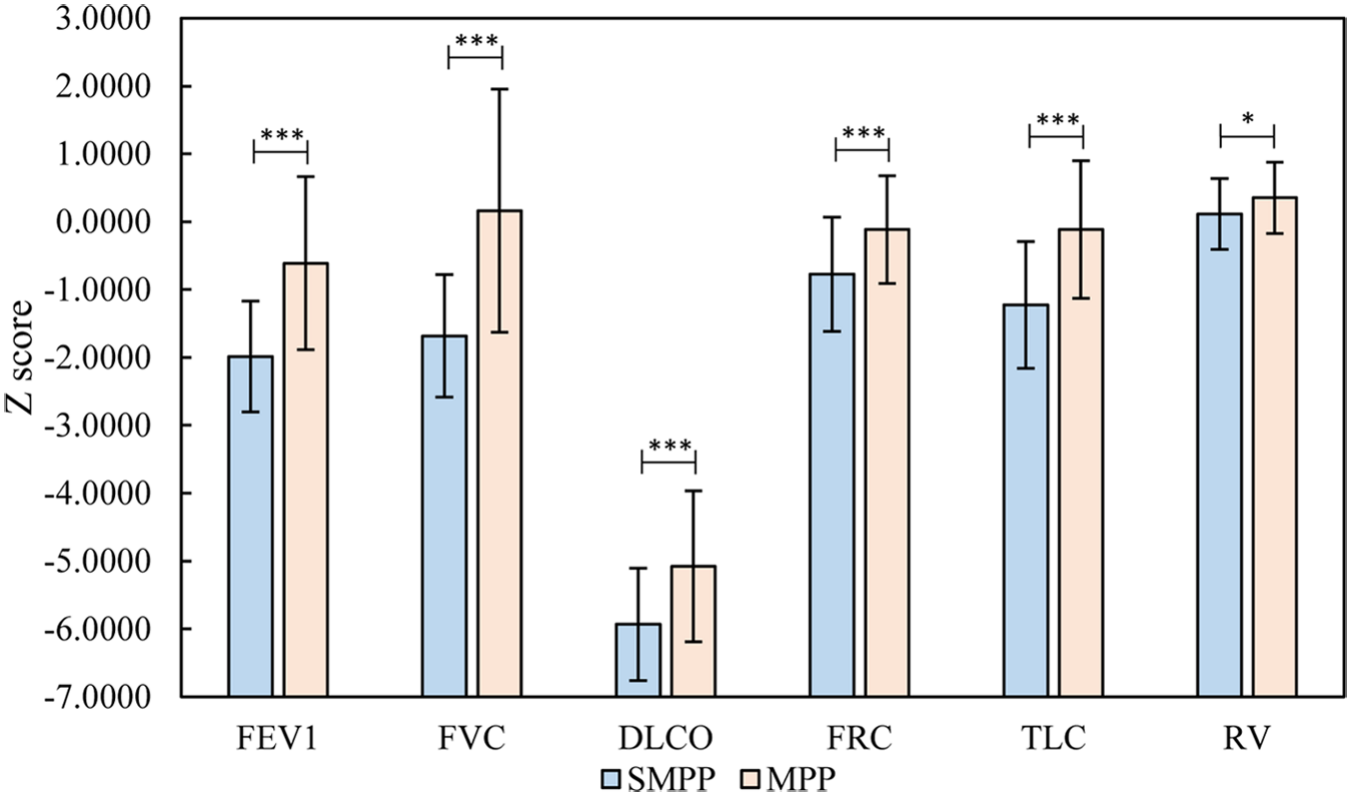
Relevant analysis of the results (FVC, FEV1/FVC, DLCO, FRC, TLC, RV) of the SMPP and MMPP groups. *P < 0.05; **P < 0.01; ***P < 0.001. MMPP group, mild *Mycoplasma pneumoniae* pneumonia group; SMPP group, severe *M. pneumoniae* pneumonia group; FEV1, forced expiratory volume in the first second; FVC, forced vital capacity; DLCO, carbon monoxide diffusion volume; FRC, functional residual capacity; TLC, total lung volume; RV, residual volume.

The results of the *Z*-scores, transformed from the raw pulmonary ventilation function and lung diffusion function data, in the two groups are shown in Table 1. In the MMPP group, all the indexes were higher than normal except for the reduction in DLCO, while in the SMPP group, FVC, FEV1, FEF75, MMEF, and DLCO were lower than normal. FVC, FEV1, FEF75, and DLCO in the SMPP group were −1.684 ± 0.902, −1.986 ± 0.818, −2.006 ± 1.258, and −5.931 ± 0.827, which were lower than −0.6104 ± 1.276, 0.1642 ± 1.795, −1.5294 ± 1.0193 and −5.078 ± 1.113 in the MMPP group, respectively, *P* < 0.05 (Figure 1). These results suggested that the MMPP group had diffuse dysfunction and the SMPP group had restrictive ventilation function reduction and diffuse dysfunction. However, there was no statistically significant difference in FEV1/FVC and MMEF between the two groups.

## 4 Discussion

Our study found that DLCO in the MMPP and SMPP groups was lower than the normal value, and DLCO in the SMPP group was significantly lower than that in the MMPP group. None of the children with SMPP had a decrease in blood oxygen saturation, but the DLCO of these children was significantly lower than the normal value. This indicates that there was abnormal gas exchange before the clinical manifestations of obvious hypoxia in these children, but it may still have been in the compensatory period. In addition, we also found that FVC, FEV1, and FEF75 in the SMPP group were lower than normal values and significantly lower than those in the MMPP group. FEV1/FVC were in the normal range, suggesting that the SMPP group had restrictive ventilation function reduction and this was more serious than that in the MMPP group. The FEF75 and MMEF in the SMPP group were lower than normal values, suggesting that SMPP can cause large and small airway dysfunction.

Respiratory system infection is a common disease in childhood. Lower respiratory tract infection, represented by pneumonia, is the main cause of death in children under 5 years old (9). MPP is one of the most common types of pneumonia in children. *M. pneumoniae* infection accounts for 10%–40% of the pathogens that cause community-acquired pneumonia (2) and up to 50% (3) in outbreak years. In recent years, the incidence of SMPP has increased year by year. During the COVID-19 pandemic, the incidence of *M. pneumoniae* infection decreased significantly but it has increased significantly again since 2023 (8). In some cities in China, children with *M. pneumoniae* infection accounted for nearly 25% of respiratory tract infections, of which SMPP accounted for 19.5%–23.1% (10). Another study showed that nearly 40% of children with MPP in China will develop SMPP (11) despite appropriate antimicrobial treatment, which poses a serious threat to these children's lives and health. Pneumonia in young children may increase the risk of asthma and impaired lung function (12). Childhood pneumonia is a major risk factor for adult chronic obstructive pulmonary disease (13). In addition, there are also relevant studies that show that MP infection is closely related to childhood bronchial asthma and is a significant cause of bronchial asthma (14–16). Therefore, detecting the changes in pulmonary function in children with MPP is helpful for the early detection of the disease. This study further explored the changes in pulmonary diffusion function after *M. pneumoniae* infection by examining the pulmonary ventilation and pulmonary diffusion functions of children with MPP, comparing the pulmonary diffusion and ventilation functions of children with SMPP and MMPP, and exploring their clinical significance.

This study found that FVC, FEV1, and FEF75 in the SMPP group were lower than normal values and significantly lower than those in the MMPP group, suggesting that the SMPP group had restrictive ventilation function reduction and this was more serious than the MMPP group. The FEF75 and MMEF in the SMPP group were lower than normal, suggesting that SMPP can cause large and small airway dysfunction, which is consistent with Yong et al. (17). This may be due to the specific antigen to *Mycoplasma* stimulating the body to produce inflammatory mediators, thus causing airway smooth muscle spasm and increased vascular permeability, leading to mucosal edema and increased secretion of mucus. This then causes congestion, edema, and erosion of tissues, reducing lung compliance, increasing elastic resistance, and reducing pulmonary surfactant, thus causing airway stenosis. The increase in airway resistance decreases the ventilation function of the large and small airways (18). A study on bacterial pneumonia in children showed that FEV1/FVC in severe pneumonia was 60.74 ± 2.83%, and the FEV1% was 60.29 ± 2.64%, which were significantly lower than the levels of the mild and control groups. However, the study did not provide information on small airway function (19).

Pulmonary diffusion function refers to the ability of certain alveolar gases to diffuse from the alveolar cavity to the capillaries and then through the alveolar-capillary membrane to the blood and combine with Hb (20). In clinical practice, the one-breath lung carbon monoxide diffusion function test (the DLCO single breath method, DLCO-SB), established by Ogilvie et al. (21), is the most commonly used. Common reasons for a decline in pulmonary diffusion function include (1) a reduction of the alveolar membrane area due to consolidation, atelectasis, or lobectomy; (2) increased alveolar membrane thickness due to pulmonary edema, formation of a transparent alveolar membrane, or pulmonary fibrosis; (3) ventilation/blood flow ratio (VA/Q) imbalance due to a pulmonary embolism, chronic obstructive emphysema, or pulmonary hypertension; and (4) a decrease in hemoglobin content due to blood system diseases, chronic renal insufficiency, or hemorrhagic anemia. Our study found that the DLCO of the SMPP group was lower than the normal value and was significantly lower than that of the MMPP group. The mechanism for this may be that MP adheres to and settles in respiratory epithelial cells after infection, damaging the cell membrane and then releasing community-acquired respiratory distress syndrome toxin (CARDs TX), hydrogen sulfide (H_2_S), hydrogen peroxide (H_2_O_2_), and other substances. The above substances cause cell dissolution, epithelial cell swelling, and necrosis (22), resulting in a reduction of the alveolar-capillary membrane area. Furthermore, after *Mycoplasma* infection, the airway ciliary activity is inhibited, and the formation of an alveolar hyaline membrane and the infiltration and edema of inflammatory cells in the alveolar wall lead to the thickening of the alveolar membrane and a decline in lung compliance (23). Some studies have shown that the airway epithelial cells are more damaged in SMPP than in MMPP (24). Furthermore, inflammatory cell infiltration is more serious in SMPP than in MMPP and there is fibrosis in the lumen and wall in the later stages, leading to airway distortion and occlusion. This may be why DLCO in the SMPP group decreased more significantly than that in the MMPP group. Marc et al. (5) also showed that 48% of the children with *M. pneumoniae* pneumonia in their study had a lower DLCO than normal. Furthermore, these children's diffuse lung function did not recover 6 months after being diagnosed with the disease. Similarly, 10% of children with COVID-19 have diffusion dysfunction, which lasts for more than 3 months, and the decline of diffusion function is more obvious in children with severe symptoms (4). Buchvald and Nielsen examined the pulmonary diffusion capacity of 20 children with hypersensitive pneumonitis and the results showed that the pulmonary diffusion capacity of these children decreased. During follow-up, it was found that the clinical symptoms of most of the children improved and their pulmonary diffusion capacity gradually recovered (25).

In addition, in our study, we found that the children with SMPP did not have a decrease in blood oxygen saturation but the DLCO of these children was significantly lower than the normal value. This indicates that there was abnormal gas exchange before the clinical manifestations of obvious hypoxia in these children but it may still be in the compensatory period when it is difficult to diagnose. A Norwegian study on COVID-19 also showed that the blood oxygen saturation of patients with severe COVID-19 was in the normal range even when their pulmonary diffusion function decreased (26). In addition, the DLCO in the SMPP group decreased more significantly than that in the MMPP group, suggesting that a decrease in DLCO can be used as an early warning indicator for the development of MMPP into SMPP. The dynamic detection of diffusion function in children may help to diagnose the disease early, improve the prognosis, and prevent the occurrence of chronic lung disease. However, this conclusion needs further verification in the future. Because most of the children in this study live in different places and lung function tests are expensive, it is difficult to dynamically monitor the prognosis of their pulmonary ventilation function and discern when their diffusion function returns to normal. However, we will attempt to follow up on the lung function of these patients in the future to obtain more data.

## Data Availability

The raw data supporting the conclusions of this article will be made available by the authors, without undue reservation.
